# A systematic review of adult pineoblastoma

**DOI:** 10.3389/fonc.2024.1442612

**Published:** 2024-12-16

**Authors:** Xiufeng Chu, Ting Zhang, Helen Benghiat, Jixuan Xu

**Affiliations:** ^1^ Department of Oncology, The Fifth Affiliated Hospital of Zhengzhou University, Zhengzhou, China; ^2^ Marshall Medical Center, The Fifth Affiliated Hospital of Zhengzhou University, Zhengzhou, China; ^3^ Hall Edwards Radiotherapy Research Group, University Hospitals Birmingham, Birmingham, United Kingdom; ^4^ Department of Gastrointestinal & Thyroid Surgery, The Fifth Affiliated Hospital of Zhengzhou University, Zhengzhou, China; ^5^ Department of Cancer Studies, University of Birmingham, Birmingham, United Kingdom

**Keywords:** pineoblastoma, adult, surgery, radiotherapy, chemotherapy, survival

## Abstract

**Background:**

Adult pineoblastoma is an extremely rare central nervous system malignancy. Limitations of tumour databases, single institution retrospective analyses and a few case reports are not sufficient to clarify treatment options. Therefore, a systematic review of comprehensive research data provides referenceable treatment options.

**Methods:**

A systematic review was performed using MEDLINE and Embase using the terms “pineoblastoma” and “adult”. Relevant articles in the references were considered to supplement this systematic review. In addition, data were analysed using Kaplan-Meier survival curves, COX analysis, chi-square tests and log-rank tests.

**Results:**

A total of 108 adult cases from 32 articles were included in this study and the median age at diagnosis was 30 years. The 5-year survival rate was 49.5% (95% confidence interval: 0.378-0.602) and the 10-year survival rate was 33.9% (95% confidence interval: 0.207-0.476). During the 10-year follow-up period, Kaplan-Meier survival curves highlighted that the gross total resection was more beneficial than subtotal resection and no surgery (P=0.018). The treatment modality of radiotherapy and chemotherapy was beneficial for survival (P<0.001; P=0.020). In addition, multivariate COX analysis showed that radiotherapy was an independent factor in the beneficial prognosis (P<0.001) and gross total resection tends to improve survival within five years (P=0.079).

**Conclusion:**

For adult pineoblastoma, gross total excision and radiotherapy can be beneficial for survival.

Systematic Review Registration: [website], identifier [registration number].

## 1 Introduction

Primary tumours of the pineal gland are rare and account for 0.1%-0.3% of intracranial malignancies (1). A variety of tumour subtypes can arise in the pineal gland. The recent World Health Organisation (WHO) Classification of Tumours of the Central Nervous System 2021 categorises primary pineal parenchymal tumours as: pineocytomas, pineal parenchymal tumours of intermediate differentiation (PPTID), pineoblastoma, papillary tumour of the pineal region and desmoplastic myxoid tumour of the pineal region, SMARCB1-mutant (2). Pineoblastoma (PB), accounts for approximately 45% of all pineal parenchymal tumour subtypes (3–5). It typically affects infants and young children with a slight female preponderance, although has been rarely reported in adults (3, 6). PB is classified as a WHO grade IV tumour and has a high rate of recurrence and propensity for spread via the cerebrospinal fluid (CSF) (7). Despite aggressive multimodality treatment, including surgery, radiotherapy and chemotherapy, the outcome of PB is poor with a 5-year survival of only 15% for patients < 5years of age (6).

Recent molecular characterisation has segregated PB into 5 molecular subgroups: PB-Group 1, PB-Group 2, PB-Group 3, RB and MYC; each with distinct clinico-pathologic and survival features (8, 9). Groups 1 to 3 PB arise in older children and adolescents and are associated with improved outcomes in contrast with patients with groups RB and MYC (9).

At present, management of adult PB is based on data extrapolated from paediatric practice. With small numbers of adult patients reported in multiple case reports and series; prognosis, as well as contribution of surgical resection and adjuvant chemo/radiotherapy on outcomes remain unclear.

### 1.1 Objectives

The objective of this study was to systematically review all adult cases of PB to determine patient characteristics as well as impact of surgical resection and adjuvant oncological therapy on prognosis from 1946 to 2021 in English journals.

## 2 Methods

This systematic review was reported as per the Preferred Reporting Items for Systematic Reviews and Meta-Analyses (PRISMA) guidelines. All articles reporting cases of pathologically confirmed adult PB were included. Using the terms “pineoblastoma” and “adult”, MEDLINE and Embase databases were searched; with results limited to those written in English and published prior to June 2021. References from searched results were used in addition, and duplicate articles removed.

Data were collected on patient and tumour baseline characteristics, overall survival and treatment received. Kaplan-Meier survival curves were used to observe unadjusted survival, and log-rank test was used to compare survival outcomes in patients who received differing surgical procedures as well as adjuvant oncological therapies. A multivariate Cox model was used to determine which clinical variables were independently related to improved survival.

The Chi-square test was used to process categorical variables. Data were analysed using SPSS version 27.0 (IBM, Armonk, New York, USA). Kaplan-Meier curves were described by STATA 16.0 (STATA corporation, College Station, TX, USA) software.

## 3 Results

### 3.1 Study selection

**Figure 1:**
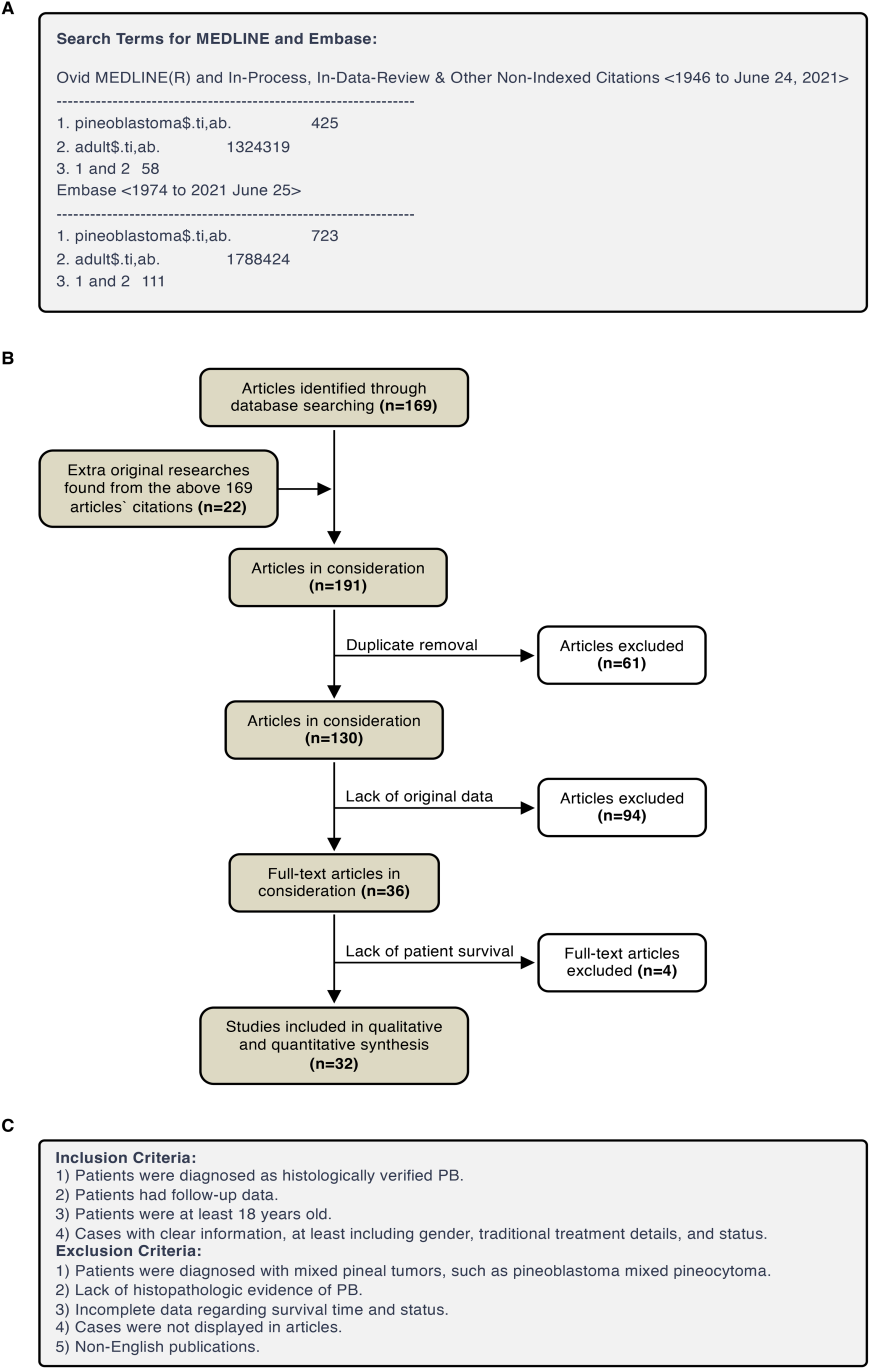
PRISMA flow diagram. **(A)** Search Terms for MEDLINE and Embase. **(B)** Eligibility assessment of papers for inclusion. **(C)** Eligibility assessment of cases for inclusion.

As shown in **Figure 1**, a total of 169 articles were identified from the MEDLINE and Embase search. Besides, during the reading-through of the content and citations of these articles, additional 22 articles were found to contain retrievable original data of adult pineoblastoma cases. Among the above 191 articles, 61 were removed because of duplication, 94 were removed for lack of original data, and 4 were removed due to lack of patient survival. In summary, a total of 32 articles (3, 10–40) were included in this systematic review, which included case reports or series with an inherent risk of bias.

For eligible cases, we extract and analyse age, gender, surgery approach (GTR/STR), radiotherapy (RT) type, RT dose, CT, CT drugs, follow-up time and status. The detail regimen of chemotherapy was not analysed because of a lack of information from most patients. From a clinical perspective, total dose of radiotherapy to the pineal region (RTP) was analysed from RT dose data. The last follow-up time was defined as survival time.

### 3.2 Findings

From the selected 32 publications, 108 adult patients (age≥18 years) with pathologically confirmed PB were identified with demographic and treatment characteristics summarised in **Table 1**. Median age at diagnosis was 30 years (range 18-81). Of the 108 cases; 48 were male (44.4%) and 60 were female (55.6%). Forty-two patients (38.9%) had their presenting symptoms reported. The most common presenting symptoms included the following; alone or in combination: headache (n=31, 73.8%), visual disturbance including Parinaud’s Syndrome (n=20, 47.6%), nausea and vomiting (n=10, 23.8%), dizziness (n=7, 16.7%), limb weakness (n=6, 14.3%) and deterioration in mobility (n=5, 11.9%). Information was available for 104 patients regarding extent of the disease at the time of diagnosis. Thirty-four patients (31.5%) were reported to have disseminated disease, and 70 (64.8%) had pineal disease only. Staging information was not available for 4 patients.

**Table 1:** Study population (n=108).

Patient characteristics	
Median age (range)	30 years (18-81)
Male	48 (44.4%)
Surgery	
GTR	14 (13%)
STR	39 (36.1%)
Biopsy	54 (50%)
Not reported	1 (0.9%)
Adjuvant RT	
Yes	94 (87%)
No	14 (13%)
RT type	
CSI	51 (54.3%)
Focal	17 (18%)
Unknown	26 (27.7%)
CT	
Yes	39 (36.1%)
No	63 (58.3%)
Unknown	6 (5.6%)
Median OS (range)	59 months (25.7-176)
Median FLUT	25.5 months

n, number of patients; GTR, gross-total resection; STR, subtotal resection; RT, radiotherapy; CSI, craniospinal irradiation; CT, chemotherapy; FLUT, follow-up time; OS, overall survival.

Of the 108 cases, 14 (13%) had gross total resection (GTR), 39 (36.1%) underwent subtotal resection (STR) and 54 (50%) had a biopsy. Extent of resection was not reported in one case (0.9%). The majority of patients [94 (87%)] received adjuvant radiotherapy following surgical resection or biopsy. Of the 94 patients who received adjuvant radiotherapy, 51 (54.3%) were treated with craniospinal irradiation (CSI). Seventeen (18%) patients received focal radiotherapy, and for 26 (27.7%) no information was found regarding radiation technique.

Only 39 patients (36.1%) received adjuvant chemotherapy (CT). The chemotherapy regimen varied significantly, and prescription information was available for only 23 patients. Although the CT drug varied for almost every patient, a cisplatin-based schedule was used in the majority (60.8%). All of the 39 patients who received adjuvant CT had also received radiotherapy. No information was available regarding toxicity of therapy.

**Figure 2:**
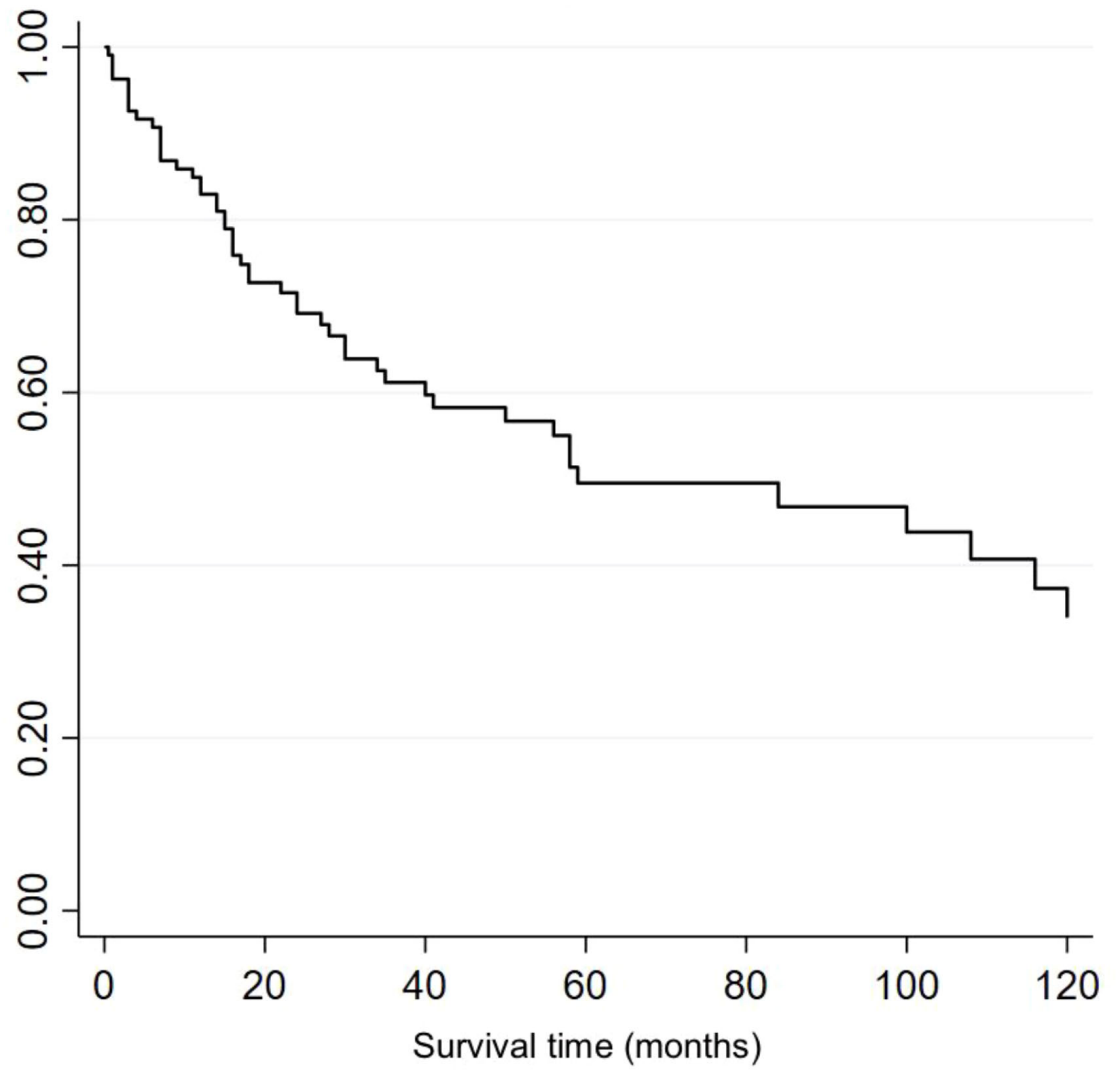
Kaplan-Meier survival curve for all patients regardless of treatment received.

Of the 108 patients, 53 (49%) patients had died. Median overall survival (OS) was 59 months, with a 5- and 10- year OS of 49.5% and 33.9% respectively (**Figure 2**). Median length of follow up was 25.5 months (range 0.5-288 months).

**Figure 3:**
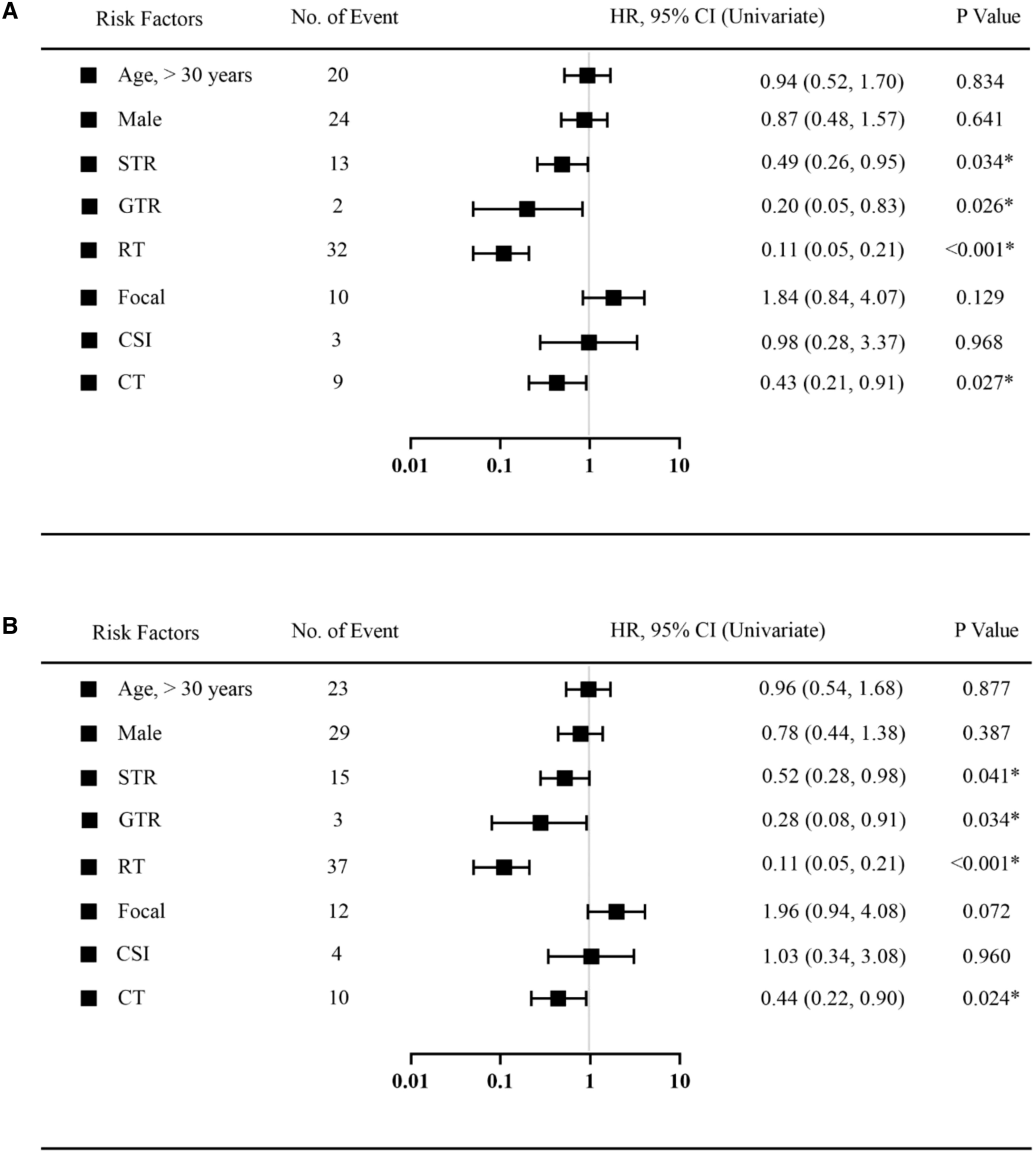
Univariate Cox regression analysis was used to estimate the prognostic factors in 5 years follow-up **(A)** or 10 years follow-up **(B)**. Black squares indicate the hazard ratio (HR). *Statistically significant. HR, hazard ratio; CI, Confidence Interval. STR, subtotal resection; GTR, gross-total resection; RT, radiotherapy; CSI, craniospinal irradiation; CT, chemotherapy.

A COX univariate analysis was used to observe and test which factors were associated with prognosis. Univariate variables that were statistically significant were included in the COX multivariate analysis model. Similar to the Kaplan–Meier curve, the COX analysis model evaluated prognostic factors in five-year and ten-year periods. Regarding the COX univariate analysis, factors including age, gender, surgery, RT, RT types, and CT were calculated (**Figure 3**). As shown in both figures, extent of resection, RT and CT are significantly associated with patient prognosis at both five and ten years.

**Figure 4:**
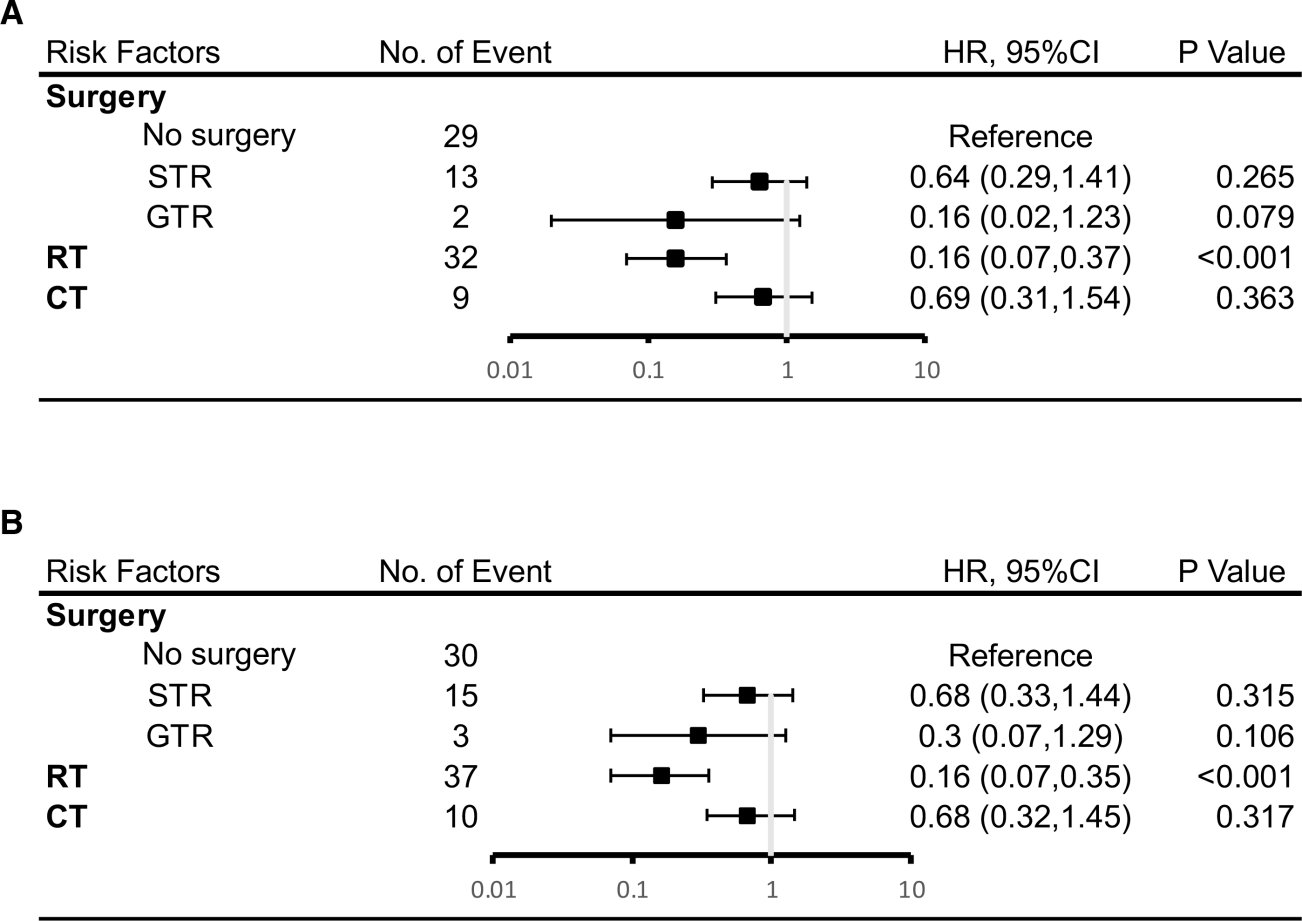
Results of multivariate analysis on overall survival at 5 years **(A)** or 10 years **(B)**. HR, hazard ratio; CI, Confidence Interval. *Statistically significant. STR, subtotal resection; GTR, gross-total resection; RT, radiotherapy; CT, chemotherapy.

As demonstrated in **Figure 4**, Cox multivariate analysis was used to determine which factors were associated with OS. There was a statistically significant benefit in OS at both 5 and 10 years for patients who received radiotherapy. (HR 0.16; p < 0.001). A trend towards improved OS at 5 years was seen for patients who had undergone a GTR (HR 0.16; p = 0.079). There was no statistically significant relationship demonstrated between the use of chemotherapy and OS.

**Figure 5:**
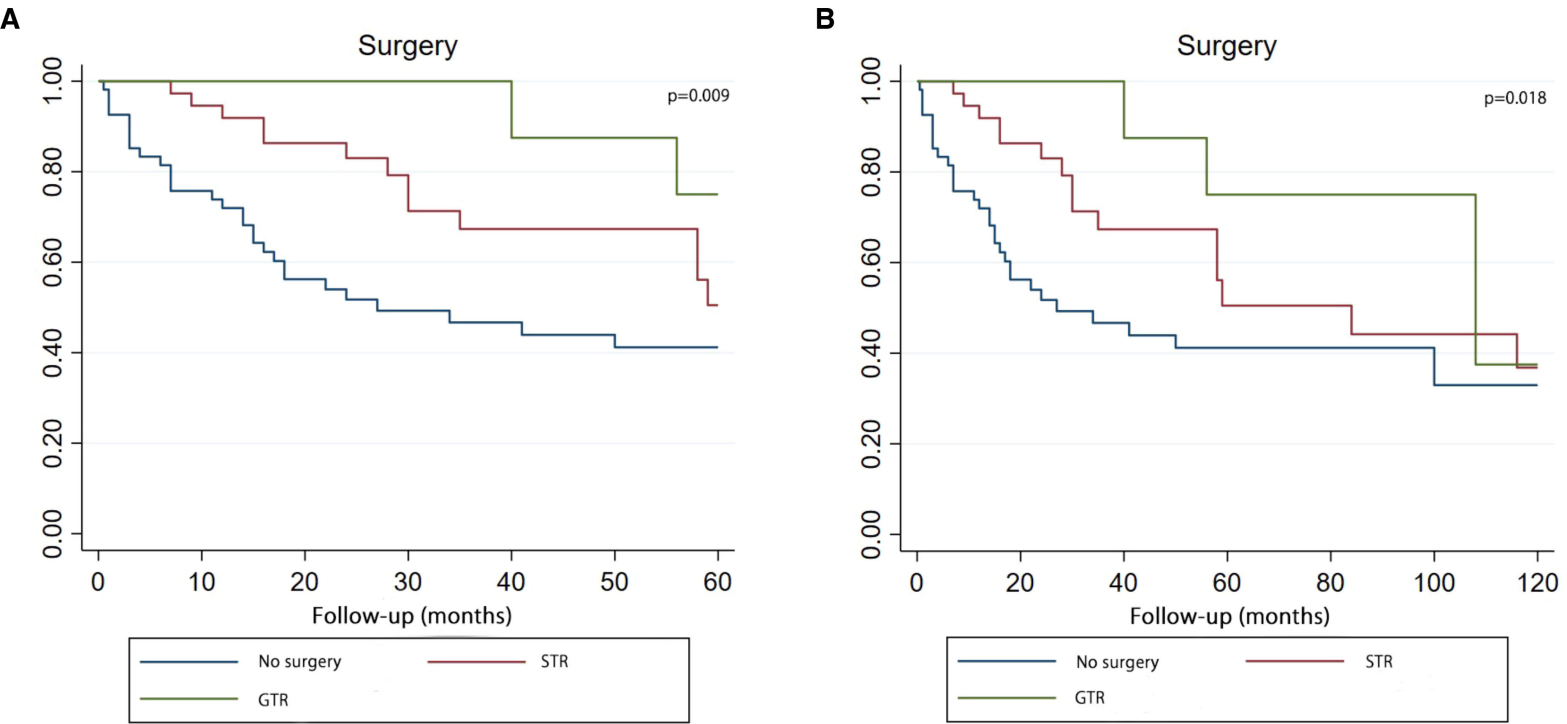
Kaplan–Meier curve analysis (Log-rank test) illustrating the survival rates of patients (n=107) between GTR, STR and no surgery for 5 years **(A)** or 10 years **(B)**. STR, subtotal resection; GTR, gross-total resection.

According to the results of COX analysis, Kaplan-Meier univariate analysis focussed on these variables: choice of surgery, RT and CT. The Kaplan-Meier survival curve demonstrated that patients who underwent surgery (whether GTR or STR) had superior overall survival at 5 and 10 years (p = 0.009, p = 0.018) (**Figure 5**).

**Figure 6:**
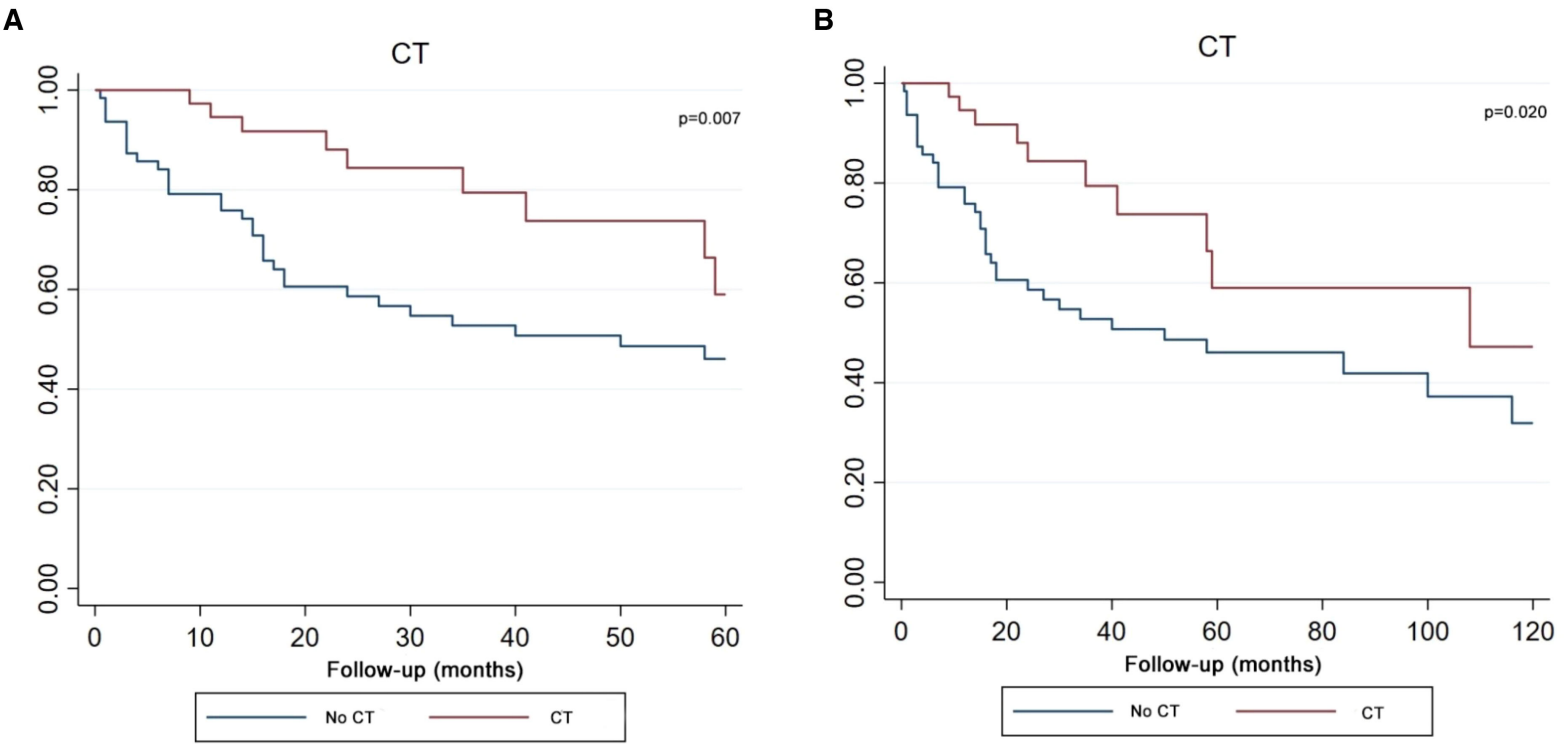
Kaplan–Meier curve analysis (Log-rank test) illustrating the survival rates of patients (n=102) between CT and no CT for 5 years **(A)** or 10 years **(B)**. CT, chemotherapy.

The Kaplan-Meier survival curve demonstrated that patients who received CT achieved better survival compared with patients who had no CT in both five-year and ten-year period time (**Figure 6**). Log-rank test P value presented that there was a statistical difference between the CT and no CT groups in two time periods (P value=0.007, P value=0.020).

**Figure 7:**
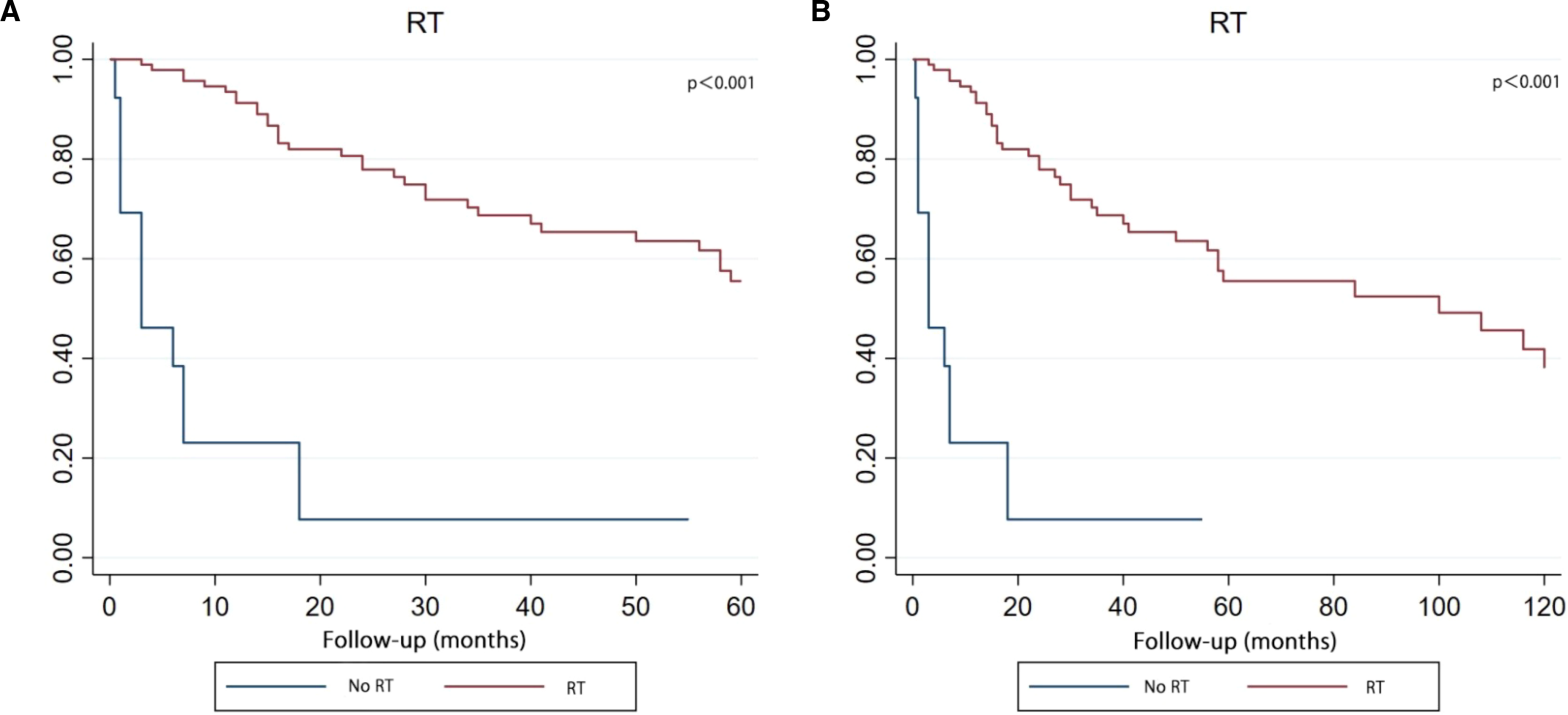
Kaplan–Meier curve analysis (Log-rank test) illustrating the survival rates of patients (n=108) between RT and no RT for 5 years **(A)** or 10 years **(B)**. RT, radiotherapy.

The Kaplan-Meier survival curve demonstrated that patients who received RT got the better survival compared with those patients who had no RT in both five-year and ten-year period time (**Figure 7**).

## 4 Discussion

### 4.1 Survival

In our study, the median survival time for this series is 59 months (range: 25.7 months – 176 months). The lowest median survival from Lee et al. was 25.7 months and the highest median survival was 176 months from Selvanathan et al. (1, 41). The large difference between the two series regarding median survival could not be analysed as neither Lee et al. nor Selvanathan et al. presented complete case data (1, 41). As the series containing the largest number of cases, Jing et al. did not provide clear survival data (42). The rest of the reference median survival range is 35-105 months (3, 10–12, 43).

However, this study is not confined to one institutional or local database and the median survival of 59 months reflects the general level of overall survival of adults with PB over the last 50 years. The 5-year survival rate for patients in this study is 49.5%, which is similar to the 5-year survival rate of 51% reported by Lutterbach et al. (12). However, Selvanathan et al. reported a 5-year survival rate of 62.8% (1). This is most likely due to the inclusion of 16- to 17- years old patients in his cases, and therefore has a greater impact on the 5-year survival rate. On every account, the prognosis for adult patients themselves is better compared to the 5-year survival rate of 15% for children aged ≤5 years (6). For this reason, younger patients with PB are more likely to develop metastases (3). Although there is currently no clear clarification of the worse prognosis in paediatric PB patients, we believe that factors such as the lack of ability to self-assess and self-care, poor medical compliance, and a weaker immune system may greatly contribute to the worse prognosis in paediatric patients compared to adults.

### 4.2 Age

Adults are defined in this study as 18 years of age or older. Furthermore, age is not a factor in the prognosis of adult PB patients. Prior to 2014, retrospective analyses of adult PB had different definitions of adult age, with some articles defining 16-year-old as adults (1, 41). Two retrospective analyses after 2015 set the age at 18 years or older and noted the difficulty of comparing clinical factors in some of the retrospective analyses because the data for patients aged 16-17 years were unclear (10, 43). In contrast, Jing et al. only included patients over 20 years of age and did not explain the specific reasons.

On the other hand, stratifying this cohort of 108 patients based on a median age of 30 years, the Kaplan-Meier curve did not find an effect of age differences on survival. Lee et al. noted that age was not a statistically significant predictor of survival (41). However, Selvanathan et al. reported that the prognosis of patients deteriorated with increasing age (1). A review of Huo et al. study found that in an overall analysis of age in 64 patients including paediatric and adult patients, the risk of survival increased with each additional year of patient age. However, when Huo et al. validated the paediatric and adult groups (age≥18 years) of the cohort separately, COX regression analysis showed that age was no longer a risk factor for both groups of patients (43). In addition, a small series of retrospective analysis of Gener et al. pointed out that age was not a risk factor for prognosis (10). Thus, Selvanathan et al. found that age was associated with prognosis, most likely because the cohort included patients under 18 years of age (1).

### 4.3 Gender

Males and females comprised 44.4% and 55.6% of the total cohort in this study, respectively. Although there were slightly more female patients than male, no gender differences were found to have an impact on improving survival rates. Most of the adult PB series display a higher proportion of female patients and no statistically significant effect of gender on prognosis (1, 10, 42, 43). Only Lee et al. stated that there was a statistical trend for gender to improve survival (41). The interpretation of this finding needs to be considered in two ways. One is that in the Lee et al. cohort, the sample size was small and predominantly male, which is not consistent with the findings of most studies. The second is that a statistical trend cannot be equated with statistical significance, and it is likely that the trend would disappear after adjusting for other factors.

### 4.4 Surgery

The prevailing surgical approach is gross total resection (GTR) and subtotal resection (STR), with GTR being the recommended approach on adult PB (44). According to the Kaplan-Meier curve, there is a significant difference in the effect of GTR, STR and no surgery on the survival rate in this study. Moreover, the effect of GTR is the best, and the effect of STR is the second. Although both GTR and STR were statistically significant in the univariate COX analysis, both lost statistical significance after the multivariate COX analysis. However, it is worth noting that the results of the multivariate COX analysis, which limited the five-year follow-up time, showed a statistical trend in GTR (P=0.079). Perhaps with an expanded sample size, GTR could be an independent variable in improving the prognosis of adult PB patients over the five years that they undergo GTR surgery. Multivariate COX analysis showed a disappearance of the tendency for GTR to improve prognosis within ten years (P=0.106), which may be related to the short survival period of the malignancy.

From a theoretical point of view, the relatively conservative approach to early surgery, such as STR, is due to the need to avoid surgical complications. With the development of clinical technology, microsurgery and neuronavigation technology can better support clinicians to choose a wider range of resection operations (6, 45). Moreover, studies have demonstrated that GTR is associated with better local control and a reduced rate of local recurrence (46, 47). Although Selvanathan et al. did not find a benefit from surgery, Tate et al. claimed that the role of GTR in the treatment of PB could not be ignored (1, 6).

### 4.5 Radiotherapy

In this study, RT not only demonstrated statistical significance in the Kaplan-Meier curve and univariate COX analysis (P<0.001), but also emerged as the only independent prognostic factor in the multivariate COX analysis. In Selvanathan et al. cohort, there was no statistical difference in survival between patients who received RT and those who did not. However, he also found that patients who received RT may have prolonged survival, acknowledging that the lack of statistical significance was due to the limitations of the sample size (1). Similarly, this issue arose in the study by Huo et al. The risk factor for RT in 14 adults with PB was protective, but not statistically significant. After he had combined the adult and paediatric samples, the prognostic impact of RT was statistically significant (43).

For the impact of the type of RT, this study attempted to explore the effect of CSI, Focal and CSI + boost on survival. Although RT type was not found a statistical difference, CSI+boost demonstrated a trend towards improved survival. In fact, there is no retrospective analysis of adult PB that explores this factor. Therefore, it is difficult to compare and validate this result. In conclusion, the prognostic impact of radiotherapy may become clearer as the sample size of future studies is expanded and more prospective trials are explored.

### 4.6 Chemotherapy

In this study, the Kaplan-Meier curve and univariate COX analysis showed that CT was beneficial and statistically significant for survival. However, a multivariate COX analysis revealed that CT could not be used as an independent prognostic variable. This may indicate that CT in combination with surgery and RT can improve survival rates. In the Tate et al. cohort, the combination of RT and CT after surgery was more beneficial to survival than the CT after surgery. However, he did not analyse the effects of CT separately nor did he distinguish between adults and children in the cohort (6). Huo et al. distinguished between adults and children and studied the prognostic impact of CT, but he did not find it to be statistically significant (43). Jing et al. found that the combination of postoperative RT and CT significantly improved survival rates (42). In clinic, one case report supported that CT was effective in clinical practice (14).

## 5 Limitations

This systematic review summarises published cases with specific data, including institutional studies and case reports. This study contains the most comprehensive number of adult PB cases available and is also the first systematic review of adult PB to provide evidence for the determination of treatment options. However, access to the database to retrieve the data was not achieved. It was also not possible to contact authors who did not provide specific data. In the study of the relationship between treatment and prognosis, the data of chemotherapeutic drugs are insufficient and cannot be statistically analysed. Sample size limitations did not allow for analysis of combination treatments. In addition, heterogeneity in tests, diagnosis and treatment modalities is objective due to differences in the year in which each patient is diagnosed. The operation of the treatment and the choice of medication are uncontrollable. However, the use of regression analysis to correct for covariates of confounding factors helped to reduce the effect of heterogeneity.

## 6 Conclusion

PB is a rare tumour of the pineal region. In adults, age and gender do not influence the overall survival of PB patients. Gross-total resection and radiotherapy are favourable factors for prognosis. Surgery combined with radiotherapy and chemotherapy is likely to be even more effective. In the future, further studies are needed to explore the contributions of radiotherapy methods, radiation doses, and chemotherapy regimens. Additionally, we advocate for the standardisation of follow-up intervals, the extension of the total duration of follow-up as well as the recording of professional activities and quality of life in original studies. Prospective studies with more restrictive selection criteria are more likely to identify the key factors that affect the survival of adult PB patients.

## Data Availability

The original contributions presented in the study are included in the article/**Supplementary Material**. Further inquiries can be directed to the corresponding authors.

## References

[1] Selvanathan SK Hammouche S Smethurst W Salminen HJ Jenkinson MD . Outcome and prognostic features in adult pineoblastomas: analysis of cases from the SEER database. Acta Neurochir (Wien). (2012) 154:863–9. doi: 10.1007/s00701-012-1330-4 22460262

[2] Louis DN Perry A Wesseling P Brat DJ Cree IA Figarella-Branger D . The 2021 WHO classification of tumors of the central nervous system: a summary. Neuro-Oncology. 23:1231–51. doi: 10.1093/neuonc/noab106 PMC8328013 34185076

[3] Farnia B Allen PK Brown PD Khatua S Levine NB Li J . Clinical outcomes and patterns of failure in pineoblastoma: a 30-year, single-institution retrospective review. World Neurosurg. (2014) 82:1232–41. doi: 10.1016/j.wneu.2014.07.010 25045788

[4] Mynarek M Pizer B Dufour C van Vuurden D Garami M Massimino M . Evaluation of age-dependent treatment strategies for children and young adults with pineoblastoma: analysis of pooled European Society for Paediatric Oncology (SIOP-E) and US Head Start data. Neuro Oncol. (2017) 19:576–85. doi: 10.1093/neuonc/now234 PMC5464312 28011926

[5] Tate MC Rutkowski MJ Parsa AT . Contemporary management of pineoblastoma. Neurosurg Clin N Am. (2011) 22:409–12, ix. doi: 10.1016/j.nec.2011.05.001 21801990

[6] Tate M Sughrue ME Rutkowski MJ Kane AJ Aranda D McClinton L . The long-term postsurgical prognosis of patients with pineoblastoma. Cancer. (2012) 118:173–9. doi: 10.1002/cncr.v118.1 21717450

[7] Tamrazi B Nelson M Blü ml S . Pineal region masses in pediatric patients. Neuroimaging Clin N Am. (2017) 27:85–97. doi: 10.1016/j.nic.2016.08.002 27889025

[8] Pfaff E Aichmü ller C Sill M Stichel D Snuderl M Karajannis MA . Molecular subgrouping of primary pineal parenchymal tumors reveals distinct subtypes correlated with clinical parameters and genetic alterations. Acta Neuropathol. (2020) 139:243–57. doi: 10.1007/s00401-019-02101-0 PMC7275775 31768671

[9] Li BK Vasiljevic A Dufour C Yao F Ho BLB Lu M . Pineoblastoma segregates into molecular sub-groups with distinct clinico-pathologic features: a Rare Brain Tumor Consortium registry study. Acta Neuropathol. (2020) 139:223–41. doi: 10.1007/s00401-019-02111-y PMC7673644 31820118

[10] Gener MA Conger AR Van Gompel J Ariai MS Jentoft M Meyer FB . Clinical, pathological, and surgical outcomes for adult pineoblastomas. World Neurosurg. (2015) 84:1816–24. doi: 10.1016/j.wneu.2015.08.005 26287970

[11] Chang SM Lillis-Hearne PK Larson DA Wara WM Bollen AW Prados MD . Pineoblastoma in adults. Neurosurgery. (1995) 37:383–90; discussion 90-1. doi: 10.1227/00006123-199509000-00003 7501100

[12] Lutterbach J Fauchon F Schild SE Chang SM Pagenstecher A Volk B . Malignant pineal parenchymal tumors in adult patients: patterns of care and prognostic factors. Neurosurgery. (2002) 51:44–55; discussion -6. doi: 10.1097/00006123-200207000-00006 12182434

[13] Fauchon F Jouvet A Paquis P Saint-Pierre G Mottolese C Ben Hassel M . Parenchymal pineal tumors: a clinicopathological study of 76 cases. Int J Radiat Oncol Biol Phys. (2000) 46:959–68. doi: 10.1016/S0360-3016(99)00389-2 10705018

[14] Gaito S Malagoli M Depenni R Pavesi G Bruni A . Pineoblastoma in adults: A rare case successfully treated with multimodal approach including craniospinal irradiation using helical tomotherapy. Cureus. (2019) 11:e5852. doi: 10.7759/cureus.5852 31754587 PMC6830851

[15] Cuccia F Mortellaro G Cespuglio D Valenti V DE Gregorio G Quartuccio E . A case report of adult pineoblastoma occurring in a pregnant woman. Anticancer Res. (2019) 39:2627–31. doi: 10.21873/anticanres.13386 31092461

[16] Neuwelt EA Glasberg M Frenkel E Clark WK . Malignant pineal region tumors. A clinico-pathological study. J Neurosurg. (1979) 51:597–607. doi: 10.3171/jns.1979.51.5.0597 501398

[17] Borit A Blackwood W Mair WG . The separation of pineocytoma from pineoblastoma. Cancer. (1980) 45:1408–18. doi: 10.1002/1097-0142(19800315)45:6<1408::AID-CNCR2820450619>3.0.CO;2-0 6986979

[18] Jooma R Kendall BE . Diagnosis and management of pineal tumors. J Neurosurg. (1983) 58:654–65. doi: 10.3171/jns.1983.58.5.0654 6834112

[19] Lesnick JE Chayt KJ Bruce DA Rorke LB Trojanowski J Savino PJ . Familial pineoblastoma. Report of two cases. J Neurosurg. (1985) 62:930–2. doi: 10.3171/jns.1985.62.6.0930 3998847

[20] Uematsu Y Itakura T Hayashi S Komai N . Pineoblastoma with an unusually long survival. Case report. J Neurosurg. (1988) 69:287–91. doi: 10.3171/jns.1988.69.2.0287 3392573

[21] Jacobs JJ Rosenberg AE . Extracranial skeletal metastasis from a pinealoblastoma. A case report and review of the literature. Clin Orthop Relat Res. (1989) 247:256–60. doi: 10.1097/00003086-198910000-00035 2676297

[22] Vaquero J Ramiro J Martí nez R Bravo G . Neurosurgical experience with tumours of the pineal region at Clinica Puerta de Hierro. Acta Neurochir (Wien). (1992) 116:23–32. doi: 10.1007/BF01541249 1319669

[23] Linggood RM Chapman PH . Pineal tumors. J Neurooncol. (1992) 12:85–91. doi: 10.1007/BF00172460 1541982

[24] Fuller BG Kapp DS Cox R . Radiation therapy of pineal region tumors: 25 new cases and a review of 208 previously reported cases. Int J Radiat Oncol Biol Phys. (1994) 28:229–45. doi: 10.1016/0360-3016(94)90162-7 8270446

[25] Mena H Nakazato Y Jouvet A Scheithauer BW . Pineoblastoma. In: Kleihues P Cavenee WK , editors. Pathology and Genetics of Tumours of the Nervous System. IARC Press, Lyon (2000). p. 115–22.

[26] Matsumoto K Higashi H Tomita S Ohmoto T . Pineal region tumours treated with interstitial brachytherapy with low activity sources (192-iridium). Acta Neurochir (Wien). (1995) 136:21–8. doi: 10.1007/BF01411431 8748823

[27] Ashley DM Longee D Tien R Fuchs H Graham ML Kurtzberg J . Treatment of patients with pineoblastoma with high dose cyclophosphamide. Med Pediatr Oncol. (1996) 26:387–92. doi: 10.1002/(SICI)1096-911X(199606)26:6<387::AID-MPO3>3.0.CO;2-D 8614374

[28] Brockmeyer DL Walker ML Thompson G Fults DW . Astrocytoma and pineoblastoma arising sequentially in the fourth ventricle of the same patient. Case report and molecular analysis. Pediatr Neurosurg. (1997) 26:36–40. doi: 10.1159/000121159 9361116

[29] Fujita A Asada M Saitoh M Nakamura H Kamikawa S Kokunai T . Pineoblastoma showing unusual ventricular extension in a young adult–case report. Neurol Med Chir (Tokyo). (1999) 39:612–6. doi: 10.2176/nmc.39.612 10487041

[30] Paulino AC Melian E . Medulloblastoma and supratentorial primitive neuroectodermal tumors: an institutional experience. Cancer. (1999) 86:142–8. doi: 10.1002/(SICI)1097-0142(19990701)86:1<142::AID-CNCR20>3.0.CO;2-Y 10391574

[31] Barlas O Bayindir C Imer M Ayan I Darendeliler E . Non-resective management of pineoblastoma. Minim Invasive Neurosurg. (2000) 43:163–70. doi: 10.1055/s-2000-14509 11108118

[32] Nakamura M Saeki N Iwadate Y Sunami K Osato K Yamaura A . Neuroradiological characteristics of pineocytoma and pineoblastoma. Neuroradiology. (2000) 42:509–14. doi: 10.1007/s002349900243 10952183

[33] Charafe-Jauffret E Lehmann G Fauchon F Michiels JF Paquis P Maraninchi D . Vertebral metastases from pineoblastoma. Arch Pathol Lab Med. (2001) 125:939–43. doi: 10.5858/2001-125-0939-VMFP 11419982

[34] Schild SE Scheithauer BW Haddock MG Wong WW Lyons MK Marks LB . Histologically confirmed pineal tumors and other germ cell tumors of the brain. Cancer. (1996) 78:2564–71. doi: 10.1002/(SICI)1097-0142(19961215)78:12<2564::AID-CNCR16>3.0.CO;2-U 8952565

[35] Schild SE Scheithauer BW Schomberg PJ Hook CC Kelly PJ Frick L . Pineal parenchymal tumors. Clinical, pathologic, and therapeutic aspects. Cancer. (1993) 72:870–80. doi: 10.1002/1097-0142(19930801)72:3<870::AID-CNCR2820720336>3.0.CO;2-X 8334641

[36] Ito T Kanno H Sato K Oikawa M Ozaki Y Nakamura H . Clinicopathologic study of pineal parenchymal tumors of intermediate differentiation. World Neurosurg. (2014) 81:783–9. doi: 10.1016/j.wneu.2013.02.007 23396072

[37] Mena H Rushing EJ Ribas JL Delahunt B McCarthy WF . Tumors of pineal parenchymal cells: a correlation of histological features, including nucleolar organizer regions, with survival in 35 cases. Hum Pathol. (1995) 26:20–30. doi: 10.1016/0046-8177(95)90110-8 7821912

[38] Ai P Peng X Jiang Y Zhang H Wang S Wei Y . Complete regression of adult pineoblastoma following radiotherapy: A case report and review of the literature. Oncol Lett. (2015) 10:2329–32. doi: 10.3892/ol.2015.3574 PMC4580009 26622845

[39] Stoiber EM Schaible B Herfarth K Schulz-Ertner D Huber PE Debus J . Long term outcome of adolescent and adult patients with pineal parenchymal tumors treated with fractionated radiotherapy between 1982 and 2003–a single institution's experience. Radiat Oncol. (2010) 5:122. doi: 10.1186/1748-717X-5-122 21184689 PMC3019157

[40] Gadish T Tulchinsky H Deutsch AA Rabau M . Pinealoblastoma in a patient with familial adenomatous polyposis: variant of Turcot syndrome type 2? Report of a case and review of the literature. Dis Colon Rectum. (2005) 48:2343–6. doi: 10.1007/s10350-005-0201-y 16400511

[41] Lee JY Wakabayashi T Yoshida J . Management and survival of pineoblastoma: an analysis of 34 adults from the brain tumor registry of Japan. Neurol Med Chir (Tokyo). (2005) 45:132–41; discussion 41-2. doi: 10.2176/nmc.45.132 15782004

[42] Jing Y Deng W Zhang H Jiang Y Dong Z Fan F . Development and validation of a prognostic nomogram to predict cancer-specific survival in adult patients with pineoblastoma. Front Oncol. (2020) 10:1021. doi: 10.3389/fonc.2020.01021 32793463 PMC7393244

[43] Huo XL Wang B Zhang GJ Ma JP Wang L Zhang LW . Adverse factors of treatment response and overall survival in pediatric and adult patients with pineoblastoma. Cancer Manag Res. (2020) 12:7343–51. doi: 10.2147/CMAR.S258476 PMC7443452 32884348

[44] Palled S Kalavagunta S Beerappa Gowda J Umesh K Aal M Abdul Razack T . Tackling a recurrent pinealoblastoma. Case Rep Oncological Med. (2014) 2014:135435. doi: 10.1155/2014/135435 PMC4158562 25210636

[45] Reddy AT Janss AJ Phillips PC Weiss HL Packer RJ . Outcome for children with supratentorial primitive neuroectodermal tumors treated with surgery, radiation, and chemotherapy. Cancer. (2000) 88:2189–93. doi: 10.1002/(SICI)1097-0142(20000501)88:9<2189::AID-CNCR27>3.0.CO;2-G 10813733

[46] Tomita T McLone DG Yasue M . Cerebral primitive neuroectodermal tumors in childhood. J Neurooncol. (1988) 6:233–43. doi: 10.1007/BF00163707 3066855

[47] Frost PJ Laperriere NJ Wong CS Milosevic MF Simpson WJ Pintilie M . Medulloblastoma in adults. Int J Radiat Oncol Biol Phys. (1995) 32:951–7. doi: 10.1016/0360-3016(94)00612-o 7607969

