# Supplementary material for: A systematic review of adult pineoblastoma
Source: Front Oncol. 2024 Dec 16;14:1442612. doi: 10.3389/fonc.2024.1442612 (PMC11683066; doi:10.3389/fonc.2024.1442612)
Supplement: Supplementary file 1 [file Table1.docx]

**Extended Figure 1. OS alterations of adult pineoblastoma over time.**

# Appendices

## Appendix 1. Study Selection Sheets for Embase and MEDLINE from Full Text

| Number | Author | Inclusion | Inclusion | Inclusion | Inclusion | Exclusion | Exclusion | Exclusion | Exclusion | Exclusion | Included or exclude paper |
| --- | --- | --- | --- | --- | --- | --- | --- | --- | --- | --- | --- |
|  |  | histologically verified PB | patients had follow-up data | Age≥18 years | clear information | mixed pineal tumours | lack of histopathologic evidence | lack of survival time and status | undisplayed data in paper | non-English publication |  |
| 1 | [Uppal DK](https://ovidsp.dc1.ovid.com/ovid-a/ovidweb.cgi?&S=MGANFPIEDBACENACKPOJKEHOPPJAAA00&Search+Link=%22Uppal+DK%22.au.&Counter5=CRS_author%7c34030077%7cprmz%7cmedline%7cprem) | NO | YES | YES | YES | YES | YES | NO | NO | NO | EXCLUDED |
| 2 | Jing Y | YES | NO | YES | YES | NO | NO | NO | YES | NO | EXCLUDED |
| 3 | Huo XL | YES | NO | YES | YES | NO | NO | NO | YES | NO | EXCLUDED |
| 4 | Bhat AR | YES | NO | YES | YES | YES | NO | NO | YES | NO | EXCLUDED |
| 5 | Mascarenhas L | NO | NO | NO | YES | YES | NO | NO | YES | NO | EXCLUDED |
| 6 | de Kock L | YES | NO | YES | NO | NO | NO | YES | YES | NO | EXCLUDED |
| 7 | Lim JX | NO | YES | YES | YES | NO | YES | NO | NO | NO | EXCLUDED |
| 8 | Pfaff E | YES | NO | YES | YES | NO | NO | NO | YES | NO | EXCLUDED |
| 9 | Cuccia F | YES | YES | YES | YES | NO | NO | NO | NO | NO | INCULDED |
| 10 | Verma A | YES | NO | YES | YES | NO | NO | NO | YES | NO | EXCLUDED |
| 11 | Endersby R | YES | NO | NO | NO | NO | NO | YES | YES | NO | EXCLUDED |
| 12 | Snuderl M | YES | NO | YES | NO | NO | NO | YES | YES | NO | EXCLUDED |
| 13 | Schultz KAP | YES | NO | NO | NO | NO | NO | YES | YES | NO | EXCLUDED |
| Number | Author | Inclusion | Inclusion | Inclusion | Inclusion | Exclusion | Exclusion | Exclusion | Exclusion | Exclusion |  |
|  |  | histologically verified PB | patients had follow-up data | Age≥18 years | clear information | mixed pineal tumours | lack of histopathologic evidence | lack of survival time and status | undisplayed data in paper | non-English publication | Included or exclude paper |
| 14 | Patel PG | YES | NO | NO | YES | NO | NO | YES | YES | NO | EXCLUDED |
| 15 | Schultz KAP | YES | NO | YES | NO | NO | NO | YES | YES | NO | EXCLUDED |
| 16 | Mynarek M | YES | NO | YES | YES | NO | NO | NO | YES | NO | EXCLUDED |
| 17 | Raleigh DR | YES | NO | YES | YES | NO | NO | NO | YES | NO | EXCLUDED |
| 18 | Gaito S | YES | YES | YES | YES | NO | NO | NO | NO | NO | INCULDED |
| 19 | Pearson AD | YES | NO | NO | YES | NO | NO | NO | YES | NO | EXCLUDED |
| 20 | Ichise H | NO | NO | NO | NO | YES | YES | YES | YES | NO | EXCLUDED |
| 21 | Gener MA | YES | YES | YES | YES | NO | NO | NO | NO | NO | INCULDED |
| 22 | Choudhri AF | YES | YES | NO | YES | NO | NO | NO | NO | NO | EXCLUDED |
| 23 | Jouvet A | YES | NO | NO | NO | NO | YES | YES | YES | NO | EXCLUDED |
| 24 | Cosman R | YES | NO | YES | NO | NO | NO | YES | YES | NO | EXCLUDED |
| 25 | Friedrich C | YES | NO | YES | YES | NO | NO | YES | YES | NO | EXCLUDED |
| 26 | Ai P | YES | YES | YES | YES | NO | NO | NO | NO | NO | INCULDED |
| 27 | Park JH | NO | YES | YES | YES | YES | YES | NO | NO | NO | EXCLUDED |
| 28 | Palled S | YES | YES | NO | YES | NO | NO | NO | NO | NO | EXCLUDED |
| 29 | Selvanathan SK | YES | YES | YES | YES | NO | NO | NO | YES | NO | EXCLUDED |
| 30 | Stoiber EM | YES | YES | YES | YES | NO | NO | NO | NO | NO | INCULDED |
| 31 | DeBoer R | YES | YES | YES | NO | NO | NO | NO | NO | NO | EXCLUDED |
| 32 | Jain N | YES | NO | YES | NO | NO | NO | YES | YES | NO | EXCLUDED |
| 33 | Gill P | YES | YES | YES | YES | NO | NO | NO | YES | NO | EXCLUDED |
| Number | Author | Inclusion | Inclusion | Inclusion | Inclusion | Exclusion | Exclusion | Exclusion | Exclusion | Exclusion |  |
|  |  | histologically verified PB | patients had follow-up data | Age≥18 years | clear information | mixed pineal tumours | lack of histopathologic evidence | lack of survival time and status | undisplayed data in paper | non-English publication | Included or exclude paper |
| 34 | Phi JH | NO | NO | NO | NO | NO | YES | YES | YES | NO | EXCLUDED |
| 35 | Pusztaszeri M | NO | YES | YES | YES | NO | YES | NO | NO | NO | EXCLUDED |
| 36 | Constantine C | NO | YES | YES | YES | NO | YES | NO | NO | NO | EXCLUDED |
| 37 | Burzynski SR | YES | YES | NO | YES | NO | NO | NO | YES | NO | EXCLUDED |
| 38 | Lee JY | YES | YES | YES | YES | NO | NO | NO | YES | NO | EXCLUDED |
| 39 | Fischer B | YES | YES | YES | YES | YES | NO | NO | NO | NO | EXCLUDED |
| 40 | Yurtseven T | YES | NO | NO | NO | NO | NO | YES | YES | NO | EXCLUDED |
| 41 | Gururangan S | YES | YES | YES | NO | YES | NO | YES | NO | NO | EXCLUDED |
| 42 | Lutterbach J | YES | YES | YES | YES | NO | NO | NO | NO | NO | INCULDED |
| 43 | Krinke GJ | NO | NO | NO | NO | YES | YES | YES | YES | NO | EXCLUDED |
| 44 | Tsumanuma I | YES | YES | NO | YES | NO | NO | NO | NO | NO | EXCLUDED |
| 45 | Fujita A | YES | NO | YES | NO | NO | NO | NO | NO | NO | EXCLUDED |
| 46 | Ikeda J | YES | NO | YES | NO | YES | NO | YES | NO | NO | EXCLUDED |
| 47 | Cho BK | YES | NO | NO | YES | NO | NO | YES | YES | NO | EXCLUDED |
| 48 | Galanis E | NO | NO | YES | YES | YES | YES | YES | YES | NO | EXCLUDED |
| 49 | Prados MD | YES | NO | NO | YES | NO | NO | YES | YES | NO | EXCLUDED |
| 50 | Mena H | YES | YES | YES | YES | NO | NO | NO | NO | NO | INCULDED |
| Number | Author | Inclusion | Inclusion | Inclusion | Inclusion | Exclusion | Exclusion | Exclusion | Exclusion | Exclusion |  |
|  |  | histologically verified PB | patients had follow-up data | Age≥18 years | clear information | mixed pineal tumours | lack of histopathologic evidence | lack of survival time and status | undisplayed data in paper | non-English publication | Included or exclude paper |
| 51 | Tsunoda S | NO | YES | YES | YES | NO | NO | NO | NO | NO | EXCLUDED |
| 52 | Chang SM | YES | YES | YES | YES | NO | NO | NO | NO | NO | INCULDED |
| 53 | Perentes E | YES | NO | YES | NO | NO | NO | YES | YES | NO | EXCLUDED |
| 54 | Rubinstein LJ | NO | NO | NO | NO | NO | YES | YES | YES | NO | EXCLUDED |
| 55 | Becker LE | NO | NO | NO | NO | NO | YES | YES | YES | NO | EXCLUDED |
| 56 | Borit A | NO | NO | NO | NO | NO | YES | YES | YES | NO | EXCLUDED |
| 57 | Herrick MK | YES | NO | YES | NO | NO | NO | YES | YES | NO | EXCLUDED |
| 58 | Vinakurau S | YES | YES | YES | YES | NO | NO | NO | NO | YES | EXCLUDED |
| 59 | Ohara A.E | YES | YES | YES | NO | NO | NO | NO | NO | NO | EXCLUDED |
| 60 | Penney S | YES | YES | NO | YES | NO | NO | NO | NO | NO | EXCLUDED |
| 61 | Severson M.C | YES | NO | YES | NO | NO | NO | YES | NO | NO | EXCLUDED |
| 62 | Li B.K | YES | NO | YES | YES | YES | NO | YES | YES | NO | EXCLUDED |
| 63 | Lisitsa T.S | YES | NO | NO | NO | NO | NO | YES | YES | NO | EXCLUDED |
| 64 | Cordova J.S. | YES | NO | NO | NO | NO | NO | YES | YES | NO | EXCLUDED |
| 65 | Tauziede Espariat A | NO | NO | NO | NO | NO | YES | YES | YES | NO | EXCLUDED |
| 66 | Pfaff E | NO | NO | NO | NO | NO | YES | YES | YES | NO | EXCLUDED |
| 67 | Autry A | YES | YES | NO | YES | NO | NO | NO | NO | NO | EXCLUDED |
| 68 | Kaur K | YES | NO | NO | NO | NO | NO | YES | YES | NO | EXCLUDED |
| Number | Author | Inclusion | Inclusion | Inclusion | Inclusion | Exclusion | Exclusion | Exclusion | Exclusion | Exclusion |  |
|  |  | histologically verified PB | patients had follow-up data | Age≥18 years | clear information | mixed pineal tumours | lack of histopathologic evidence | lack of survival time and status | undisplayed data in paper | non-English publication | Included or exclude paper |
| 69 | Batth I | YES | NO | NO | NO | NO | NO | YES | YES | NO | EXCLUDED |
| 70 | Lee C | YES | NO | YES | YES | NO | NO | YES | YES | NO | EXCLUDED |
| 71 | Gatalica Z | YES | YES | YES | YES | YES | NO | NO | NO | NO | EXCLUDED |
| 72 | Zahedi S | NO | NO | NO | NO | NO | YES | YES | YES | NO | EXCLUDED |
| 73 | Pfaff E | NO | NO | NO | NO | NO | YES | YES | YES | NO | EXCLUDED |
| 74 | Snuderl M | YES | NO | NO | NO | NO | NO | YES | YES | NO | EXCLUDED |
| 75 | Vizcaino M.A | YES | NO | NO | NO | NO | NO | YES | YES | NO | EXCLUDED |
| 76 | Nagurney M | YES | NO | NO | NO | YES | NO | YES | YES | NO | EXCLUDED |
| 77 | Paul M. | YES | YES | NO | YES | NO | NO | NO | NO | NO | EXCLUDED |
| 78 | Sherani F. | YES | NO | NO | NO | NO | NO | YES | YES | NO | EXCLUDED |
| 79 | Hu E. | NO | NO | NO | NO | NO | YES | YES | YES | NO | EXCLUDED |
| 80 | Solomon D | YES | NO | YES | NO | YES | NO | YES | YES | NO | EXCLUDED |
| 81 | Yanagisawa T | YES | NO | NO | NO | NO | NO | YES | YES | NO | EXCLUDED |
| 82 | Lobjanidze N. | YES | NO | NO | NO | NO | NO | YES | YES | NO | EXCLUDED |
| 83 | Lulla R.R. | NO | NO | NO | NO | NO | YES | YES | YES | NO | EXCLUDED |
| 84 | Messing-Junger M. | YES | NO | NO | NO | NO | NO | YES | YES | NO | EXCLUDED |
| 85 | Foulkes W. | NO | NO | NO | NO | NO | YES | NO | NO | NO | EXCLUDED |
| 86 | Farnia B. | YES | YES | YES | YES | NO | NO | NO | NO | NO | INCULDED |
| Number | Author | Inclusion | Inclusion | Inclusion | Inclusion | Exclusion | Exclusion | Exclusion | Exclusion | Exclusion |  |
|  |  | histologically verified PB | patients had follow-up data | Age≥18 years | clear information | mixed pineal tumours | lack of histopathologic evidence | lack of survival time and status | undisplayed data in paper | non-English publication | Included or exclude paper |
| 87 | Wilcox R.E. | YES | NO | NO | NO | NO | NO | YES | YES | NO | EXCLUDED |
| 88 | De Braganca K. | YES | NO | NO | NO | NO | NO | YES | YES | NO | EXCLUDED |
| 89 | Green A.L. | NO | NO | NO | NO | NO | YES | YES | YES | NO | EXCLUDED |
| 90 | Armaghani A. | YES | YES | YES | YES | YES | NO | NO | NO | NO | EXCLUDED |
| 91 | Lulla R.R. | YES | YES | NO | YES | NO | NO | NO | NO | NO | EXCLUDED |
| 92 | Messing-Junger M. | YES | YES | NO | YES | NO | NO | NO | NO | NO | EXCLUDED |
| 93 | Hart M.G. | YES | NO | YES | NO | NO | NO | YES | YES | NO | EXCLUDED |
| 94 | Jain S. | YES | NO | NO | NO | NO | NO | YES | YES | NO | EXCLUDED |
| 95 | Singer S. | YES | NO | NO | NO | NO | NO | YES | YES | NO | EXCLUDED |
| 96 | Sugiyama K. | YES | NO | NO | NO | NO | NO | YES | YES | NO | EXCLUDED |
| 97 | Mahajan A. | YES | NO | NO | NO | NO | NO | YES | YES | NO | EXCLUDED |
| 98 | Selvanathan S.K. | YES | NO | NO | NO | NO | NO | YES | YES | NO | EXCLUDED |
| 99 | Anonymous | YES | NO | NO | NO | NO | NO | YES | YES | NO | EXCLUDED |
| 100 | Tiago M. | YES | NO | NO | NO | NO | NO | YES | YES | NO | EXCLUDED |
| 101 | Lulla R.R | YES | NO | NO | NO | NO | NO | YES | YES | NO | EXCLUDED |
| 102 | Komakula S. | NO | NO | NO | NO | NO | YES | YES | YES | NO | EXCLUDED |
| 103 | Finlay J | NO | NO | YES | NO | NO | YES | YES | YES | NO | EXCLUDED |
| Number | Author | Inclusion | Inclusion | Inclusion | Inclusion | Exclusion | Exclusion | Exclusion | Exclusion | Exclusion |  |
|  |  | histologically verified PB | patients had follow-up data | Age≥18 years | clear information | mixed pineal tumours | lack of histopathologic evidence | lack of survival time and status | undisplayed data in paper | non-English publication | Included or exclude paper |
| 104 | Sessa F. | YES | NO | NO | NO | NO | YES | NO | NO | NO | EXCLUDED |
| 105 | Bao S. | NO | NO | NO | NO | NO | YES | YES | YES | NO | EXCLUDED |
| 106 | Mader I. | YES | NO | NO | NO | NO | NO | YES | YES | NO | EXCLUDED |
| 107 | Krinke G.J | NO | NO | NO | NO | NO | YES | YES | YES | NO | EXCLUDED |
| 108 | Borit A | YES | YES | YES | YES | NO | NO | NO | NO | NO | INCULDED |

**Appendix 2. Data Extraction Sheet**

| Number | Age | Gender | Surgery | Radiotherapy | Chemotherapy | Follow-up (month) | Status | RT type | CT drugs | RT dose (Gy) |
| --- | --- | --- | --- | --- | --- | --- | --- | --- | --- | --- |
| 1  2  3  4  5  6  7  8  9  10  11 | 45  31  24  36  40  70  81  44  30  51  66 | female  female  male  female  female  female  male  female  female  female  female | STR  GTR  no surgery  no surgery  STR  no surgery  no surgery  STR  GTR  no surgery  no surgery | RT  RT  RT  RT  RT  RT  no RT  RT  RT  no RT  RT | CT  CT  CT  CT  no CT  no CT  no CT  CT  no CT  no CT  no CT | 180.0  108.0  240.0  11.0  4.0  7.0  55.0  3.0  156.0  .5  288.0 | Death  Death  survive  Death  survive  Death  survive  survive  survive  Death  Death | CSI  CSI  CSI | vincristine |  |

| Number | Age | Gender | Surgery | Radiotherapy | Chemotherapy | Follow-up (month) | Status | RT type | CT drugs | RT dose (Gy) |
| --- | --- | --- | --- | --- | --- | --- | --- | --- | --- | --- |
| 12  13  14  15  16  17  18  19  20  21  22 | 21  30  31  39  40  25  27  55  59  35  30 | female  female  female  male  male  male  male  female  male  male  male | GTR  STR  STR  STR  no surgery  STR  GTR  STR  STR  no surgery  no surgery | RT  RT  RT  RT  RT  RT  RT  RT  RT  RT  RT | CT  CT  CT  CT  CT  no CT  no CT  CT  CT  no CT  no CT | 12.0  9.0  35.0  59.0  14.0  30.0  62.0  26.0  24.0  27.0  72.0 | survive  Death  Death  Death  Death  Death  survive  survive  survive  Death  survive | CSI+boost  CSI+boost  CSI+boost  CSI+boost  CSI+boost  CSI+boost  CSI+boost  CSI+boost  CSI+boost  CSI+boost  CSI+boost | CDDP, CCNU, vincristine  lomustin, cisplatin, vincristine  lomustin,procarbazine,vincristine  lomustin,procarbazine,vincristine  lomustin, cisplatin, vincristine  lomustin, cisplatin, vincristine  lomustin, cisplatin, vincristine | 36,54  45,59  24,55  24,54  54,71  31,54  35,55  30,72  30,72  30,50  30,50 |

| Number | Age | Gender | Surgery | Radiotherapy | Chemotherapy | Follow-up (month) | Status | RT type | CT drugs | RT dose (Gy) |
| --- | --- | --- | --- | --- | --- | --- | --- | --- | --- | --- |
| 23  24  25  26  27  28  29  30  31  32  33  34 | 23  38  43  34  20  22  25  22  49  30  36  18 | female  female  female  female  female  male  male  male  male  male  female  male | no surgery  GTR  GTR  STR  STR  no surgery  no surgery  no surgery  STR  no surgery  STR  STR | RT  RT  RT  RT  RT  no RT  RT  RT  RT  RT  RT  RT | no CT  no CT  no CT  no CT  no CT  no CT  no CT  no CT  CT  CT  CT  CT | 15.0  6.0  16.0  24.0  18.0  18.0  14.0  18.0  122.0  61.0  57.0  58.0 | Death  survive  survive  Death  survive  Death  survive  survive  survive  survive  survive  Death | Focal  CSI+boost  CSI+boost  CSI+boost  CSI+boost  Focal  CSI+boost  CSI+boost  CSI+boost  CSI | cisplatinum,etoposide,ifosfamide  cisplatinum,etoposide,ifosfamide  cisplatinum,etoposide,ifosfamide  methyl-gag | 55  34,50  36,56  30,50  31,54  56  27,54  27,54  27,54  51 |

| Number | Age | Gender | Surgery | Radiotherapy | Chemotherapy | Follow-up (month) | Status | RT type | CT drugs | RT dose (Gy) |
| --- | --- | --- | --- | --- | --- | --- | --- | --- | --- | --- |
| 35  36  37  38  39  40  41  42  43  44 | 46  40  28  22  25  46  23  32  20  25 | male  female  female  female  female  male  female  female  female  female | STR  STR  STR  STR  no surgery  GTR  STR  GTR  STR | RT  RT  RT  RT  RT  RT  RT  RT  RT  RT | no CT  CT  no CT  CT  no CT  CT  CT | 36.0  121.0  182.0  44.0  17.0  36.0  18.0  120.0  56.0  30.0 | survive  Death  survive  survive  Death  survive  survive  Death  Death  Death | CSI+boost  CSI+boost  CSI+boost  CSI+boost  CSI+boost  CSI+boost  CSI+boost | cisplatin,etoposid,gemcitabine  Cisplatin and Etoposide | 34,60  35,50  36,52  32,59  36,56  36,54  40,54 |

| Number | Age | Gender | Surgery | Radiotherapy | Chemotherapy | Follow-up (month) | Status | RT type | CT drugs | RT dose (Gy) |
| --- | --- | --- | --- | --- | --- | --- | --- | --- | --- | --- |
| 45  46  47  48  49  50  51  52  53  54 | 45  18  52  29  39  21  22  19  47  29 | female  male  female  male  female  female  male  female  male  female | STR  STR  no surgery  no surgery  no surgery  no surgery  no surgery  no surgery  no surgery  no surgery | RT  RT  RT  no RT  no RT  no RT  no RT  no RT  no RT  no RT | no CT  no CT  no CT  no CT  no CT  no CT  no CT | 28.0  7.0  25.0  18.0  7.0  3.0  1.0  3.0  1.0  6.0 | Death  Death  survive  Death  Death  Death  Death  Death  Death  Death | Focal  Focal  CSI+boost |  | 58  31,54 |

| Number | Age | Gender | Surgery | Radiotherapy | Chemotherapy | Follow-up (month) | Status | RT type | CT drugs | RT dose (Gy) |
| --- | --- | --- | --- | --- | --- | --- | --- | --- | --- | --- |
| 55  56  57  58  59  60  61  62  63  64 | 52  18  23  23  23  48  18  23  23  19 | male  male  female  female  female  female  male  female  male  male | no surgery  no surgery  no surgery  STR  no surgery  STR  STR  no surgery  STR  STR | no RT  no RT  no RT  RT  RT  RT  RT  RT  RT  RT | no CT  no CT  no CT  no CT  no CT  no CT  no CT  CT  CT  CT | 7.0  3.0  1.0  18.0  100.0  84.0  58.0  32.0  20.0  16.0 | Death  Death  Death  survive  Death  Death  Death  survive  survive  survive | Focal  CSI+boost  Focal |  | 45  10,50  51 |

| Number | Age | Gender | Surgery | Radiotherapy | Chemotherapy | Follow-up (month) | Status | RT type | CT drugs | RT dose (Gy) |
| --- | --- | --- | --- | --- | --- | --- | --- | --- | --- | --- |
| 65  66  67  68  69  70  71  72  73  74 | 21  22  21  23  19  32  31  21  36  51 | male  female  male  male  male  male  male  male  female  female | STR  STR  STR  GTR  no surgery  STR  no surgery  no surgery  no surgery  STR | RT  RT  RT  RT  RT  RT  RT  RT  RT  RT | CT  CT  CT  CT  CT  no CT  no CT  no CT  no CT  no CT | 28.0  14.0  24.0  100.0  36.0  61.0  70.0  57.0  51.0  16.0 | survive  survive  survive  survive  survive  survive  survive  survive  survive  Death | CSI  CSI+boost  CSI  CSI+boost  CSI+boost  CSI+boost  Focal | etoposide, cisplatinum  ifosfamide,carboplatinum,etoposide | 30,54  36,50  36,50  36,50  60 |

| Number | Age | Gender | Surgery | Radiotherapy | Chemotherapy | Follow-up (month) | Status | RT type | CT drugs | RT dose (Gy) |
| --- | --- | --- | --- | --- | --- | --- | --- | --- | --- | --- |
| 75  76  77  78  79  80  81  82  83  84 | 49  26  18  19  49  60  57  30  35  21 | male  male  male  female  female  male  female  female  male  male | STR  STR  no surgery  no surgery  no surgery  no surgery  no surgery  no surgery  no surgery  no surgery | RT  RT  RT  RT  RT  no RT  RT  RT  RT  RT | no CT  no CT  CT  CT  no CT  no CT  no CT  no CT  no CT  CT | 12.0  108.0  24.0  24.0  125.0  3.0  4.0  16.0  14.0  15.0 | Death  survive  Death  survive  survive  Death  Death  Death  Death  survive | Focal  CSI  CSI+boost  CSI+boost  CSI+boost  Focal  CSI  CSI+boost  CSI+boost  CSI+boost | etoposide,cisplatin,epirubicin  methotrexate  cisplatinum,CCNU,vincristine | 60  38,54  36,66  36,50  56  21  34,55  36,57  50,54 |

| Number | Age | Gender | Surgery | Radiotherapy | Chemotherapy | Follow-up (month) | Status | RT type | CT drugs | RT dose (Gy) |
| --- | --- | --- | --- | --- | --- | --- | --- | --- | --- | --- |
| 85  86  87  88  89  90  91  92  93  94 | 35  31  25  24  30  18  20  44  26  19 | male  female  female  female  female  female  female  male  male  male | no surgery  no surgery  no surgery  STR  STR  no surgery  GTR  no surgery  no surgery  no surgery | RT  RT  RT  RT  RT  RT  RT  RT  RT  RT | no CT  no CT  no CT  no CT  no CT  CT  no CT  CT  no CT  no CT | 61.0  12.0  15.0  16.0  116.0  4.0  22.0  21.0  50.0  142.0 | survive  Death  Death  Death  Death  survive  survive  survive  Death  Death | CSI+boost  CSI+boost  CSI+boost  Focal  Focal  Focal  Focal  CSI+boost  Focal  Focal | etoposide,carboplatinum | 35,55  36,53  36,56  45  46  56  60  36,54  35  49 |

| Number | Age | Gender | Surgery | Radiotherapy | Chemotherapy | Follow-up (month) | Status | RT type | CT drugs | RT dose (Gy) |
| --- | --- | --- | --- | --- | --- | --- | --- | --- | --- | --- |
| 95  96  97  98  99  100  101  102  103  104 | 66  38  55  77  46  22  30  26  18  25 | female  female  female  male  male  male  female  male  female  female | no surgery  GTR  STR  no surgery  GTR  no surgery  GTR  no surgery  STR  STR | RT  RT  RT  RT  RT  RT  RT  RT  RT  RT | no CT  no CT  no CT  no CT  no CT  CT  no CT  CT  no CT  CT | 79.0  57.0  128.0  34.0  93.0  41.0  40.0  68.0  48.0  45.0 | survive  survive  survive  Death  survive  Death  Death  survive  survive  survive | Focal  Focal  CSI+boost  Focal  Focal  CSI+boost  CSI+boost  CSI+boost  CSI+boost  CSI+boost |  | 54  54  36,56  54  59  37,60  31,56  36,50  36,56  36,54 |

| Number | Age | Gender | Surgery | Radiotherapy | Chemotherapy | Follow-up (month) | Status | RT type | CT drugs | RT dose (Gy) |
| --- | --- | --- | --- | --- | --- | --- | --- | --- | --- | --- |
| 105  106  107  108 | 42  32  35  20 | female  male  female  female | GTR  no surgery  no surgery  no surgery | RT  RT  RT  RT | CT  no CT  CT  CT | 11.0  77.0  22.0  18.0 | survive  survive  Death  survive | CSI+boost  CSI+boost  CSI+boost  CSI+boost | cisplatinum,CCNU,vincristine  Cytoxan,etoposide  etoposide,cisplatinum,hydroxyurea | 36,54  36,54  36,56  36,53 |

GTR, gross total resection; STR, subtotal resection; RT, radiotherapy; CT, chemotherapy; CSI, Cranial spinal irradiation.
